# The Medical Professions after the War

**Published:** 1918-08-10

**Authors:** 


### THE EDITOR'S LETTER-BOX.

### The Medical Professions after the War.

### SOME GLOOMY FOREBODINGS OF A PRACTITIONER.

*To the Editor of* The Hospital.

Sir,—Every grade of workers is preparing to help itself in that period vaguely named "after the war"; every grade of worker has helped itself in that definite period "the war" excepting only the medical professions. The miner, the ironworker, the agricultural labourer, the solicitor, the retailer, the wholesaler, all by combination, cunning, and insistence have raised their wages, increased their profits, improved their financial position. Why? Because they all have sense, comradeship, and combination. The only profession that does not share now in the good things going, and will not share in the future, is the profession of healing. Will not share? Yes, will not share, unless the various branches combine, organise, and insist on their share. Doctors and nurses have been sweated in the past, and will be sweated in the future, unless they organise themselves, stand shoulder to shoulder, and inform the sweaters—that is, the remainder of the community—that those who look after the sick must be paid, like the rest of the community, what they can extort from the community for their services, just as the miner, the railway man, the solicitor, the retailer, or the teacher gets, and proudly takes and pockets, what he can get from the community. Let us be done with "noble professions," "self-sacrificing women," "healing hands," and all such twaddle, and get to business and make a good living like the rest. A lucky nurse gets about the pay of a third-rate cook. The average earnings of surgeons and pork-butchers compared would reveal that the surgeon is paid about a quarter the receipts of the butcher. Or, if you think that comparison "derogatory to the dignity of the noble profession of medicine," try comparison with the Civil Service; its certainty and its pensions.

Whether we like it or no, the time has come when we must combine, must make a great and strong trade union, and call it a trade union, and enforce its rules as fiercely—yes, fiercely—as any trade union of them all. If not, we are going to be sweated. Labour is going to have a great say in the future of Great Britain, and the one thing Labour will not do is to pay its servants if its servants are not combined to enforce payment. See how the great co-operative societies, societies of "working men," "Labour," treat the men and women who work for them. No bloated capitalistic shopkeeper is so merciless and hard.

No, unless the doctor is prepared to be sweated on about the wage a third-class mechanic will refuse, and the nurse is willing to work at a wage a scullery-maid will command, doctors and nurses must have their trade union. It need not concern the few medical men who can demand fees of hundreds of guineas, nor the few fashionable nurses who can get what they like to ask, but the rank and file. They are the men and women who must combine and make a fund, and strike for their own hand as regardless of the sufferings of the rest of the community as the dock labourer, the teacher, the miner, or the 'bus-driver. If they do not, the community will take advantage of them and laugh at them for fools.

As to the coroners! What does it matter that they gas from their chairs? It is the heartless brute of a doctor if the doctor refuses attendance till he sees his fee. It is never the heartless brute of a baker or butcher who won't supply bread or meat without cash down. It is never the heartless brute of a neighbour who runs for the doctor but won't put his hand in his pocket for a fee for a sick friend. Let the coroners preach, and laugh at them. Preaching is what they are paid for, and they being solicitors in the main, it will be a shrewd thrust to ask "How often do you work for a doubtful client without security for fees?" "Do you act gratis for the orphan and the widow?"

Should it be one union or several? Probably several, with strong amalgamation and a central committee. Doctors in one, nurses in another, chemists in a third, instrument-makers in a fourth, and so on. But all amalgamated, working together, able to put the screw on together and get good value for the services of each and all. Either we are human beings out to make a good living individually, to have a high standard of living, plenty to eat and to drink, and the wherewithal to be merry; or we are a philanthropic society. We can't be both. It is hypocrisy to pretend to be both or the last. So an amalgamated society of medical professions, or, if you prefer, a religious order, vows of poverty, obedience, chastity, and service; a gown and a cord and an obscure grave.—Yours faithfully,

Exasperated.

